# Preclinical evaluation of a bivalent conjugate vaccine against *Salmonella* Typhi and Paratyphi A

**DOI:** 10.3389/fimmu.2026.1726455

**Published:** 2026-05-01

**Authors:** Renzo Alfini, Roberta Di Benedetto, Martina Carducci, Vikram Paradkar, Hanuman Mallubhotla, Santosh Renukuntla, Dharmeshkumar Patel, Senthilkumar Manoharan, Mallikarjuna Panchakshari, Mahesh Kyasani, Nupur Sengupta, Laura B. Martin, Omar Rossi, Simona Rondini, Carlo Giannelli, Francesca Micoli

**Affiliations:** 1 GSK Vaccines Institute for Global Health, Siena, Italy; 2 Biological E Limited, Hyderabad, India

**Keywords:** 3-deoxy-D-manno-oct-2-ulosonic acid (Kdo) linkage, conjugation chemistry, glycoconjugate, *Salmonella* Paratyphi A, *Salmonella* Typhi, vaccine

## Abstract

**Introduction:**

*Salmonella enterica* serovars Typhi and Paratyphi A cause millions of cases and thousands of deaths annually, predominantly affecting children in South and Southeast Asia. Following the licensure and World Health Organization (WHO) prequalification of a typhoid conjugate vaccine (TCV), efforts have focused on developing a bivalent vaccine incorporating an *S.* Paratyphi A glycoconjugate component to increase vaccine coverage and protection against enteric fever.

**Methods:**

To create the bivalent vaccine, the *S.* Paratyphi A serovar-specific O-antigen (O:2) was conjugated to the CRM_197_ carrier protein using different chemistries. 1−Cyano−4−dimethylaminopyridinium tetrafluoroborate (CDAP) chemistry was selected based on its simplicity in manufacturing, stability data, and immunogenicity observed in preclinical studies. The final bivalent formulation combined Vi–CRM_197_ (TCV) for *S.* Typhi and O:2–CRM_197_ for *S.* Paratyphi A. Immunogenicity was evaluated in animal models.

**Results:**

The newly developed bivalent vaccine was stable over time, when stored at 2–8 °C. A slight increase of free O:2 was detected, but remained below 15% after 1 year of storage. In preclinical models, the bivalent formulation elicited strong immune responses against both antigens, higher than the corresponding unconjugated polysaccharides. These results have guided the selection of the formulations to be tested in clinical studies.

**Conclusions:**

This vaccine has the potential to broaden protection against enteric fever, particularly in high-incidence regions. Further clinical trials are ongoing to confirm efficacy and safety in humans.

## 1 Introduction

Enteric fever remains an important cause of morbidity and mortality in developing countries, particularly in Asia, where a high prevalence of severe disease is recorded among children and adolescents (1). In Africa and the Americas, enteric fever is almost exclusively caused by *Salmonella enterica* serovar Typhi (2–4). However, in Asia, a large proportion of cases are caused by *S*. Paratyphi A. Globally, 14.3 million cases of typhoid and paratyphoid fevers occurred in 2017 (5), with *S*. Typhi is responsible for 11 million cases of typhoid fever (76.3% of cases of enteric fever) and more than 135,000 deaths worldwide (6, 7).

Over the past three decades, age-standardized incidence, mortality, and disability-adjusted life year (DALY) rates for enteric fever have declined globally, with multiple Global Burden of Disease (GBD) analyses reporting negative annualized percentage changes from 1990 to 2021. Despite these improvements, the absolute burden remains substantial in low- and middle-income countries (LMICs). Burden is geographically concentrated, particularly in parts of sub-Saharan Africa and South Asia (8–10). Children and young adults account for a large share of deaths and DALYs worldwide; GBD estimates indicate that combined typhoid and paratyphoid continue to cause millions of cases and substantial DALYs among children under 15 (11).

Regionally, sub-Saharan Africa consistently ranks among the areas with the highest incidence, mortality, and DALY rates for combined typhoid and paratyphoid (8–10). *Salmonella* Paratyphi A is prevalent in Asia, responsible for up to 35% of all enteric fever episodes in India and Nepal, and more than 60% in China (12). Its high incidence in young children and the difficulty in clinically distinguishing paratyphoid from typhoid fever support the development of a bivalent vaccine covering both *Salmonella* Typhi and Paratyphi A, particularly for South and Southeast Asia.

Another concern supporting the development of a bivalent vaccine is the growing antimicrobial resistance (AMR) of *Salmonella*, which leads to increased difficulty in treatment (9). A total of 1.27 million deaths were attributable to AMR in 2019, with a high burden in LMIC (13). An extensive study based on literature screening between 1990 and 2018 (12) showed that AMR of *Salmonella* Typhi and Paratyphi A is worsening. AMR in *S.* Typhi is commonly associated with the H58 lineage that emerged spontaneously in India in 1987 and became radially distributed throughout South Asia and then globally in the ensuing years (14). Many investigations have documented a growing problem of antimicrobial resistance among *Salmonella* in endemic areas, with fluoroquinolone resistance particularly common (15). Some reports indicate ciprofloxacin resistance rates in parts of South Asia approaching 20%. In 2020, extensively drug-resistant (XDR) typhoid represented approximately 70% of cases in Pakistan, and ciprofloxacin resistance in South Africa exceeded 85% (16). In the European Union, *Salmonella* isolates from human infections have shown substantial resistance to ampicillin (≈29.8%), sulfonamides (≈30.1%), and tetracyclines (≈31.2%) (17). Moreover, international travel, cross-border trade, and geographic spread have contributed to the introduction of multidrug-resistant *Salmonella* into previously non-endemic regions (18).

Currently, multiple licensed and commercially available conjugate vaccines against typhoid fever are available, composed of the polysaccharide Vi linked to various carrier proteins. Vi is a linear homopolymer of α-1,4-N-acetylgalactosaminouronic acid, 60%–90% O-acetylated at the C-3 position (19, 20) capable of conferring protection against *S.* Typhi infection (21). In 2017, Vi conjugated to tetanus toxoid (TT) was prequalified by the World Health Organization (WHO) (Typbar TCV, Bharat Biotech International Ltd., India) (22, 23). Another Vi–TT typhoid conjugate vaccine (TCV), ZyvacTcv-PFS (Zydus Lifesciences, India) (24), and a Vi–diphtheria toxoid (DT) conjugate, SKYTyphoid [SK Bioscience (South Korea)] (25), were prequalified by WHO in 2024. Bio-TCV, a Vi-DT, developed by Bio Farma (Indonesia), was licensed in 2023 by the Indonesian Food and Drug Authority and is seeking WHO prequalification.

Vi polysaccharide from *Citrobacter freundii*, conjugated to the nontoxic mutant of diphtheria toxin, CRM_197_, developed by GSK Vaccine Institute for Global Health (GVGH) and subsequently transferred to Biological E Ltd. (India), was licensed and WHO prequalified in 2020 (TYPHIBEV) (20, 26). This conjugate is based on the derivatization of CRM_197_ with adipic acid dihydrazide (ADH) and conjugation to Vi polysaccharide (Vi–CRM_197_) by carbodiimide chemistry (20).

With the idea of combining a typhoid conjugate with the *S*. Paratyphi A component, different strategies are being evaluated. The most advanced approaches use the *S*. Paratyphi A serovar-specific O-antigen (OAg, O:2) as a target antigen. O:2 has been described as both an essential virulence factor and a protective antigen for *S*. Paratyphi A (27, 28). O:2 consists of a trisaccharide backbone composed of rhamnose (Rha), mannose (Man), and galactose (Gal), with a branch of paratose (Par) from the C-3 of Man (conferring factor 2 serogroup specificity) and a branch of glucose (Glc) from the C-6 of Gal; C-3 of Rha is partially O-acetylated (29). O:2–TT conjugates were previously shown to be safe and to elicit anti-O:2 IgG antibodies in different age groups (28). The Serum Institute of India is advancing the development of a combination of O:2–DT with A Vi–TT glycoconjugate, after a successful Phase 1 study (30). The addition of a novel *S*. Paratyphi A conjugate component to an already licensed TCV is the strategy employed by SK Bioscience in collaboration with IVI, combining SKYTyphoid vaccine with *S*. Paratyphi A O:2–DT (31). Boston Children’s Hospital has instead proposed the use of MAPS technology, utilizing biotin–rhizavidin interactions for linking polysaccharide and protein, for a combination of Vi and O:2 with promising preclinical results (32).

GVGH, in partnership with Biological E, is developing a bivalent conjugate vaccine that combines TYPHIBEV (Vi–CRM_197_) with a novel *S.* Paratyphi A conjugate using the same carrier protein. In this study, several conjugation chemistries were evaluated to produce the *S.* Paratyphi A component. A random conjugation approach using 1−cyano−4−dimethylaminopyridinium tetrafluoroborate (CDAP) activation of the O:2 polysaccharide was chosen, based on animal immunogenicity and stability data. The selected O:2 construct was formulated together with Vi–CRM_197_ into a bivalent vaccine, with and without aluminum hydroxide adjuvant. Immunogenicity results in mice and rabbits were encouraging and supported the selection of the bivalent vaccine formulations to progress into a Phase 1 clinical trial (NCT05613205).

## 2 Materials and methods

### 2.1 Synthesis of Vi–CRM_197_

Synthesis, purification, and characterization of the Typhoid Vi–CRM_197_ glycoconjugate was performed as published by Arcuri et al. (33). Polysaccharide (fragmented Vi, average MW 43 kDa) was dissolved at 50 mg/mL in 100 mM MES, pH 6.0. NHS and then EDAC were added to have 0.33 M NHS and EDAC/Vi repeating units with a molar ratio of 5. ADH−derivatized protein was (20) then added to reach 7.8 mg/mL Vi in 20 mM MES pH 6 and incubated 2 h at room temperature. A Vi-to-protein weight ratio of 1:1 was used. The conjugate was purified by tangential flow filtration.

### 2.2 Synthesis and characterization of O:2–ADH, O:9–ADH, and O:4,5–ADH intermediates

Derivatization of the OAg (*Salmonella* Paratyphi A O:2, *Salmonella* Enteritidis O:9, and Typhimurium O:4,5) with ADH was performed as already reported (34). OAg was solubilized in 100 mM AcONa, pH 4.5, at a concentration of 20–40 mg/mL. ADH and NaBH_3_CN were added as solids, both with a ratio of 1.2∶1 by weight with respect to the OAg. The solution was mixed at 30 °C for 1 h. The reaction mixture was desalted against water on a G-25 column.

The TNBS colorimetric method was used for total NH_2_ group quantification. The calibration curve was built with ADH in the range 24–200 nmol NH_2_/mL. Samples/standards (500 μL) were added to 500 μL of a 4% NaHCO_3_ solution followed by 500 μL of 0.1% TNBS solution. Samples/standards were vortexed and heated at 40 °C for 2 h. After this, adsorption at 335 nm was read.

Analysis for the determination of free ADH was performed eluting all OAg–ADH samples on a Tosoh TSKgel 3000 PWXL column with 0.1 M NaH_2_PO_4_, 0.1 M NaCl, and 5% CH_3_CN, pH 7.2, at a flow rate of 0.5 mL/min. Sample volume of injection was 80 µL. Free ADH was detected at 214 nm against a standard curve of ADH in the range 5–100 nmol/mL.

### 2.3 Synthesis of O:2–ADH–SIDEA–CRM_197_

Synthesis of O:2–ADH–SIDEA–CRM_197_ was performed as already published (34, 35). The O:2–ADH intermediate was synthesized as previously described. For introduction of the second linker adipic acid bis-(N-hydroxysuccinimide) ester (SIDEA), O:2–ADH (50 mg/mL) was dissolved in water/DMSO (1:9, v/v). After complete solubilization of the polysaccharide, triethylamine (TEA) was added (molar ratio TEA:total NH_2_ groups = 5; total NH_2_ groups include both the phosphoethanolamine groups on the OAg and the hydrazide groups introduced with the first linker), followed by SIDEA (molar ratio SIDEA:total NH_2_ groups = 12). The reaction mixture was stirred at room temperature for 3 h. The reaction mixture was added to a volume of 100 mM citrate buffer, pH 3 (equal to twice the reaction volume), and mixed at 4 °C for 30 min. Under these conditions, unreacted SIDEA was precipitated and was removed by centrifugation. The O:2–ADH–SIDEA was recovered from the supernatant by precipitation with ethanol (final ethanol concentration, 80%). The pellet was washed twice with 100% ethanol (1.5 volumes relative to the reaction mixture volume for each wash) and then lyophilized. For conjugation to CRM_197_, O:2–ADH–SIDEA was dissolved in a 100 mM NaH_2_PO_4_ buffer, pH 7.2, and CRM_197_ was added to a final protein concentration of 20 mg/mL, with a final buffer capacity of 100 mM and a molar ratio of active ester groups to CRM_197_ of 30:1. The reaction was mixed at room temperature for 2 h. The conjugate was purified using hydrophobic interaction chromatography (HIC) to separate unreacted O:2 polysaccharide and CRM_197_ protein. Post chromatography, the O:2–CRM conjugate was concentrated and diafiltered with phosphate buffered saline solution using tangential flow filtration. Subsequently, the purified and formulated conjugate was sterile filtered and stored at 2–8°C.

### 2.4 Synthesis of O:2–CDAP–CRM_197_

CDAP was added to O:2 at 10 mg/mL in saline solution with a weight ratio of CDAP to O:2 of 0.3. pH was adjusted to 9 by adding TEA and the reaction was incubated at room temperature for 3 min. Then, CRM_197_ was added with a protein-to-O:2 weight ratio of 1 to achieve chemical conjugation. The reaction was conducted at room temperature for 3 h, maintaining the pH at 9. After completion of conjugation, glycine 1 M was added to quench the conjugation reaction (overnight at 2–8 °C). The reaction mixture was then concentrated using ultrafiltration and then the conjugate O:2–CRM was purified using HIC to separate unreacted O:2 polysaccharide and CRM_197_ protein. Post chromatography, the O:2–CRM conjugate was concentrated and diafiltered with phosphate buffered saline solution using tangential flow filtration. Subsequently, the purified and formulated conjugate was sterile filtered and stored at 2–8°C.

### 2.5 Synthesis of O:2ox–CRM_197_

Synthesis of the O:2ox–CRM_197_ conjugate was performed with a similar protocol reported in (36). Lyophilized O:2 was solubilized in 100 mM AcONa buffer, pH 5, at 10 mg/mL and then added with NaIO_4_ solution (3.75 mM final concentration). The mixture was stirred for 2 h in the dark. To quench residual sodium periodate, a solution of Na_2_SO_3_ phosphate buffer, pH 7.2, was added to the oxidized O:2 mixture. Conjugation of O:2ox to CRM_197_ was performed in 100 mM phosphate buffer, pH 7.2, at a final concentration of O:2 and CRM_197_ of 10 and 5 mg/mL, respectively.

NaBH_3_CN was added as powder soon after (O:2:NaBH_3_CN = 1:1 in weight), and the reaction was kept overnight at 37 °C in an oven. Residual aldehyde groups were quenched with the addition of NaBH_4_ (O:2:NaBH_4_ = 1:1 in weight). The mixture was kept at room temperature for 2 h. Conjugate was purified by HIC on a Phenyl HP column (GE Healthcare). The purified conjugate was sterile filtered and stored at 2–8°C.

### 2.6 O:2–ADH–SIDEA–CRM_197_, O:2–CDAP–CRM_197_, and O:2ox–CRM_197_ conjugate characterization

For all conjugates, total saccharide was quantified by high-performance anion-exchange chromatography with pulsed amperometric detection (HPAEC-PAD) (36), total protein content was quantified by micro BCA [using BSA as standard and following the manufacturer’s instructions (Thermo Scientific)], and the ratio of saccharide to protein was calculated. Conjugation efficiency and conjugate aggregation were verified by HPLC-SEC of the conjugation crude material versus free CRM_197_ (36).

Free O:2 was determined by HPAEC-PAD after conjugate precipitation with deoxycholate (34). The conjugate diluted to 1.5 mL with NaCl 0.1 M at 100 μg/mL in protein was added with 0.15 mL of deoxycholate 1% solution. The mixture was chilled in ice for 30 min and added to 75 μL of cooled HCl 1 M. The solution was mixed and the pellet was removed by centrifugation (12,000 rcf for 30 min at 4 °C). Free O:2 was assayed in the supernatant. Alternatively, a disposable solid-phase extraction C4 column was used (29, 37). One milliliter of conjugate, diluted to 100 μg/mL (protein basis) in 5 mM sodium phosphate, pH 7.2, containing 150 mM NaCl and 0.005% Tween 20, was applied to a Vydac Bioselect C4 solid-phase extraction cartridge. The sample was allowed to pass through by gravity, and the flow-through was collected. Then, 1 mL of 20% acetonitrile with 0.05% TFA was loaded onto the column to maximize recovery of free polysaccharide; this eluate was combined with the initial flow-through. The combined–mL fraction was dried in a centrifugal evaporator and reconstituted in an appropriate volume of water for quantification of free saccharide by HPAEC-PAD.

Free CRM_197_ content was quantified by HPLC-SEC. Ten microliters of conjugate was injected on a Tosoh TSKgel 2000 SW-XL column using as eluent 0.1 M Na_2_SO_4_/0.1 M sodium phosphate, pH 6.6, at 0.3 mL/min. Unconjugated CRM_197_ was quantified using a fluorescence detector (excitation 280 nm/emission 336 nm) with a calibration curve ranging from 10 to 100 μg/mL CRM_197_.

O-acetyl (OAc) % was calculated by micro-Hestrin colorimetric assay performed on a plate. The conjugate was diluted to 0.5 mg/mL expressed as O:2. Sample aliquots were also used to prepare blanks. A total of 114 μL of sample, standard, or blank was loaded per well. Samples and blanks were analyzed in triplicate. A standard curve of acetylcholine was prepared in the range 50–805 nmol/mL. To each well, in the order listed, was added 36 μL of basic hydroxylamine solution (hydroxylamine hydrochloride 4 M diluted 1.46× with 50% NaOH), 50 μL of 4 M HCl, and 50 μL of 0.37 M ferric chloride (prepared in 0.1 M HCl). For blank wells, 4 M HCl was added before the basic hydroxylamine. Any bubbles on the plate were removed by centrifugation. An additional centrifugation step was performed to remove the precipitate formed during the reaction. The post-centrifugation supernatant was transferred to a new plate and the absorbance was measured at 540 nm.

### 2.7 Vi–CRM_197_ + O:2–CRM_197_ bivalent formulations

Vi–CRM_197_ and O:2–CRM_197_ were diluted at the desired concentration with phosphate buffer 10 mM, pH 7.2–7.5. All solutions were previously sterile filtered 0.22 μm. In the case of adsorbed bivalent formulations on aluminum hydroxide, it was added at this point in a quantity foreseen by the immunization scheme continuing stirring up to 60–90 min at room temperature.

### 2.8 Characterization of bivalent formulations

The characterization here reported was applied to both the adsorbed and unabsorbed formulations.

pH measurement was performed following the Ph. Eur. Method, while osmolality was measured by an osmometer.

Vi polysaccharide quantity was estimated by HPAEC-PAD analysis as previously reported (33). In the case of formulation with aluminum hydroxide, to assess unabsorbed Vi, formulations were centrifuged at 12,000 rpm for 10 min at 4 °C and collected supernatants were subjected to centrifugal evaporation drying. The day after, samples were reconstituted in water.

To determine the percentage of free Vi polysaccharide, the same procedure described for Vi content (HPAEC-PAD) analysis was used, performed after protein (conjugate) precipitation with deoxycholate in acidic conditions, as reported above for O:2 (34).

Total O:2 content was determined by a phenol-sulfuric assay (36) that quantifies the total amount of detectable sugar in the sample as hexose weight equivalent (glucose calibration curve). The glucose calibration curve covered 62.5–250 µg/mL. Two hundred microliters of each sample or standard was transferred to tubes and 200 µL of 5% phenol solution was added, followed by 1 mL of sulfuric acid. After a 10-min incubation at room temperature, the tubes were vortexed and, following a further 30-min incubation, absorbance was read at 490 nm. For the determination of the % of adsorption, formulations were centrifuged at 12,000 rpm for 10 min at 4 °C and collected supernatants were subjected to centrifugal evaporation drying before reconstitution in water for analysis.

To determine the percentage of free O:2, the same procedure described for total O:2 content (phenol sulfuric colorimetric assay) was used, performed after protein (conjugate) precipitation with deoxycholate in acidic conditions.

### 2.9 Animal studies

All animal sera used in this study were derived from immunization experiments performed at the Charles River Laboratories (France). The animal studies were reviewed by the local ethical committee (project codes: for mice, APAFIS #2017122815209931 v5, date of approval: 28 March 2018; for rabbits, APAFIS #2016061011167092, date of approval: 11 September 2018) and carried out in compliance with animal welfare standards according to European Directive 63/2010, the local legislation, and the GSK policy on the Care, Welfare and Treatment of Animals. Animals were randomized at their arrival at the animal facility. Prior to conducting animal studies, based on historical variability data, the number of animals per group was calculated to detect at least a fourfold difference in IgG geometric mean response with 80% power (Student’s *t*-test, α = 0.05). Following immunization, animals were monitored once daily on the day of immunization and for the subsequent 2 days to assess general health parameters, such as fur condition and mobility, as well as to evaluate the injection site for signs of inflammation or other reactions. During all studies performed, no signs of toxicity were observed. The operators who performed the immunizations were blinded to the immunization schedules.

In the study comparing the two bivalent formulations, Vi–CRM_197_ + O:2–ADH–SIDEA–CRM_197_ and Vi–CRM_197_ + O:2–CDAP–CRM_197_, 10 BALB/c female mice per group were injected subcutaneously (SC) with 50 μL of 1.25 μg of O:2 and 1.25 μg of Vi on days 0 and 28 with an aluminum hydroxide concentration of 1.5 mg/mL Al^3+^.

In the case of the *in vivo* study conducted to investigate the impact of aluminum hydroxide concentration in the bivalent formulation, eight female CD-1 wild-type mice per group were injected intraperitoneally (IP) with 100 μL/dose of 2.5 μg of O:2 and 2.5 μg of Vi on days 0 and 28 with an increasing concentration of aluminum hydroxide (0–0.25–0.75–1.5 mg/mL Al^3+^). One group of mice was vaccinated with O:2 and Vi unconjugated polysaccharide at a dose of 5 μg each with 1.5 mg/mL Al^3+^.

In the case of the rabbit study, eight female (>1.5 kg) New Zealand White rabbits per group were injected intramuscularly (IM) with 500 μL/dose of 25 μg of conjugated O:2 and 25 μg of conjugated Vi on days 0 and 28 with an increasing concentration of aluminum hydroxide (0–0.25–0.75–1.5 mg/mL Al^3+^).

In all animal studies, blood was collected on day 27 (post I), with final bleed on day 42 (post II). The collected blood samples were left at room temperature for 30 min and centrifuged at 2,800 rpm for 15 min at +5 °C ± 3 °C, and sera were collected.

### 2.10 Immunoassay methods

Anti-O:2 and anti-Vi IgG responses were measured by enzyme-linked immunosorbent assay (ELISA), while functionality of the antibodies was evaluated by serum bactericidal activity (SBA) against *S.* Paratyphi A.

NuncMaxisorp 384-well round bottom (Nunc) plates were coated with O:2 at a final concentration of 2 μg/mL in Carbonate buffer (pH 9.6) or Vi in PBS at a final concentration of 1 μg/mL and incubated at 4 °C overnight (16 ± 2 h); then, coating solution was aspirated (without wash) and plates were blocked with 5% PBS milk for 1 h at 25 °C; after three washes with PBS-Tween 0.05%, primary antibodies, diluted 1:100, 1:4,000, and 1:160,000 in 5% PBS milk for rabbit sera or 0.1% BSA PBS-Tween for mouse sera, were added and incubated for 2 h at 25 °C. Each serum dilution was prepared as a single sample that was assayed in triplicate, with triplicates on different ELISA plates. Plates were then washed three times with PBS-Tween 0.05% and incubated for 1 h at 25 °C with alkaline phosphatases conjugated secondary antibodies (Sigma) diluted 1:4,000 and 1:10,000 for Vi and O:2 for mice, and 1:10,000 both for Vi and O:2 for rabbits, in 0.1% BSA PBS-Tween. Then, plates were washed again three times with PBS-Tween, and p-Nitrophenyl phosphate substrate (Sigma-fast, Sigma-Aldrich, Massachusetts, USA) was added and incubated for 1 h at 25 °C. Absorbances at 405 and 490 nm were acquired using an automatic plate reader (Biotek). Ten dilution points (twofold steps apart) of anti-antigen-specific standard serum at defined ELISA units/mL (EU/mL) were assayed in duplicate on each ELISA plate, together with four blank wells used as negative control on the ELISA plate and as additional standard points. One ELISA unit (EU) is defined as the reciprocal of the dilution of the standard serum that yields an absorbance of 1 in the standard assay. Antigen-specific antibody concentrations in the samples are expressed as EU/mL obtained from the lowest serum dilution out of the ones tested for which OD falls within the linear range of the standard curve. Values are reported as the average (from the triplicates) of the EU/mL values obtained by interpolating sample OD values against the calibrated standard curve, taking into account the initial serum dilution.

For SBA, individual animal sera and pooled hyperimmune standard sera were heat inactivated (HI) at 56 °C for 30 min prior to being tested in a serum bactericidal assay based on luminescent readout (38) against *S.* Paratyphi A NVGH308 (39). SBA was performed in 96-well round-bottom sterile plates (Corning). Dilutions of HI test sera plus a negative control sample with no sera were incubated for 3 h at 37 °C in the presence of 20% exogenous complement [baby rabbit complement (BRC)] and bacteria cells (approximately 200,000 CFU/mL) in 100 μL of reaction mix volume/well. At the end of the incubation, the plates were centrifuged for 10 min at 4,000 *g*, the supernatant was discarded to remove ATP derived from dead bacteria, and live bacterial pellets resuspended in PBS were transferred to a white round-bottom 96-well plate (Greiner) and mixed 1:1 (vol/vol) with BacTiter-Glo reagent (Promega). The reaction mixture was incubated for 5 min at room temperature in an orbital shaker, and the luminescence signal was measured using a luminometer. A four-parameter non-linear regression was applied to raw luminescence for all the serum dilutions tested, including the negative control well that was assigned a reading equivalent to 10^15^ sera dilution. SBA titers are reported as IC_50_, defined as serum dilutions giving 50% inhibition of the ATP level in the negative-control well. Titers below the minimum measurable level of luminescence were arbitrarily given an IC_50_ of half of the first dilution of sera tested. GraphPad Prism 7 software (GraphPad Software) was used for fitting and IC_50_ determination.

### 2.11 Statistical analysis

Stability data trends and impact of different aluminum hydroxide doses on immune response were verified applying linear regression analysis (respectively vs. time or aluminum hydroxide doses), checking the significance of the slopes (Minitab Software). Comparisons of IgG or SBA titers between two groups were performed with the two-tailed Mann–Whitney test, and comparisons across multiple groups used the Kruskal–Wallis test followed by Dunn’s post-hoc test (GraphPad).

## 3 Results

### 3.1 Evaluation of different chemistries for the conjugation of O:2 to CRM_197_ carrier protein

**Figure 1:**
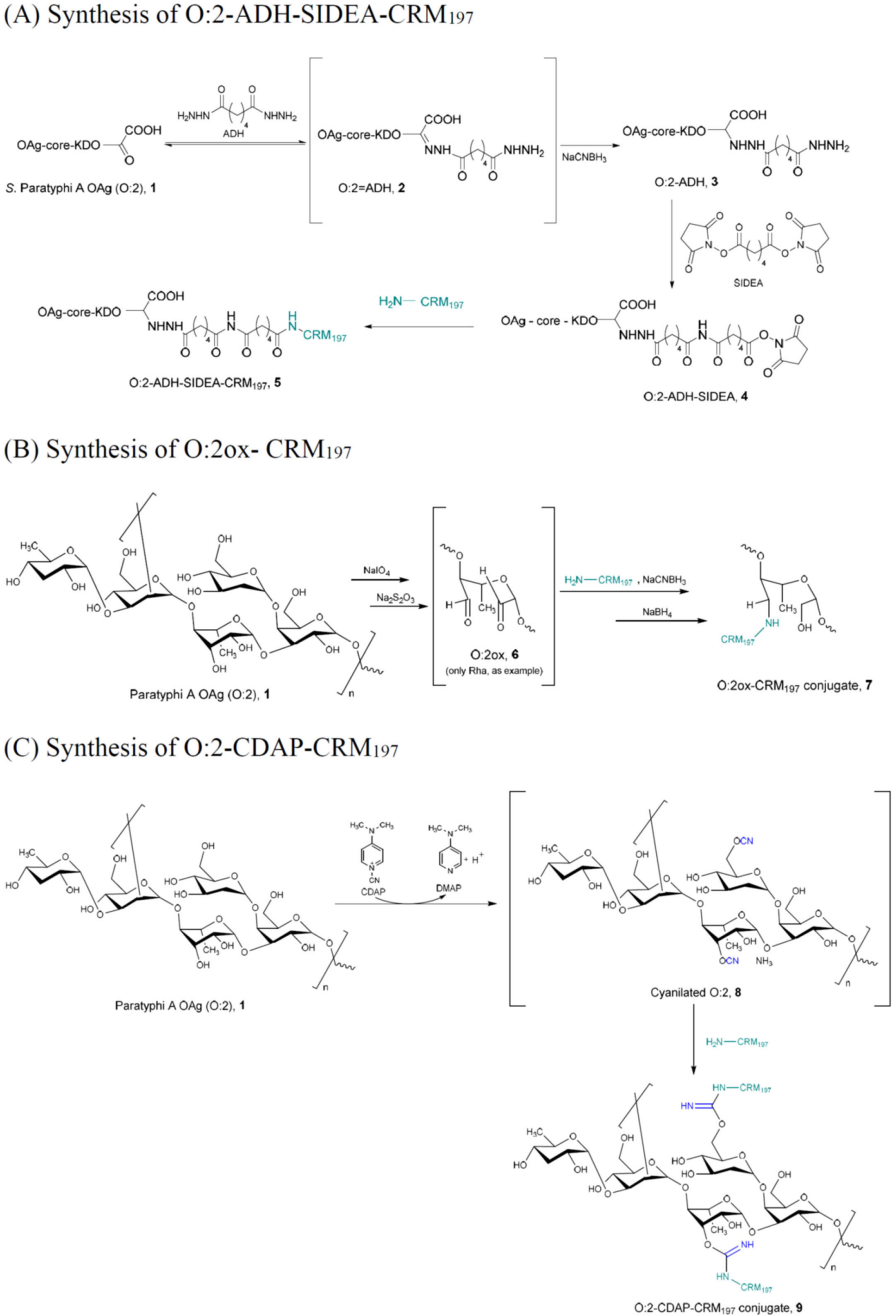
Schematics of conjugation chemistry approaches to produce O:2–CRM_197_. **(A)** Synthesis of the O:2–ADH–SIDEA–CRM_197_ conjugate (5) through the selective chemistry approach involving the terminal Kdo sugar of the O:2 OAg core region. The overall process is based on three steps: (1) formation of the O:2–ADH (3) intermediate through reductive amination of the terminal Kdo sugar with ADH and sodium cyanoborohydride (NaCNBH_3_); (2) synthesis of the intermediate O:2–ADH–SIDEA (4) based on the introduction of reactive esters by reaction with SIDEA; and (3) linkage to CRM_197_ carrier protein leading to the selective conjugate (5), involving NH_2_ groups most likely belonging to lysine residues of the protein. **(B)** Synthesis of the O:2ox–CRM_197_ conjugate (7) by random oxidation of the O:2 followed by reductive amination with CRM_197_. After random oxidation of the sugar with sodium periodate followed by quenching of the excess of NaIO_4_ with sodium thiosulfate Na_2_S_2_O_3_, oxidized O:2 (O:2ox, 6) is added with NaCNBH_3_ and CRM_197_. Finally, residual unreacted aldehyde groups are quenched with NaBH_4_, leading to the final random conjugate O:2ox–CRM_197_ (7). **(C)** Synthesis of the O:2–CDAP–CRM_197_ glycoconjugate (9): hydroxyl groups along the OAg chains are randomly activated through cyanylation with CDAP (8). Then, CRM_197_ carrier protein is added, leading to the final random O:2–CDAP–CRM_197_ conjugate (9).

The selective conjugation strategy reported in **Figure 1A** consists of the linkage of O:2 chains to lysines on the carrier protein through the terminal 3-Deoxy-D-manno-oct-2-ulosonic acid (Kdo) sugar of the OAg core (36, 40). This chemistry is able to generate a well-defined conjugate without impacting the OAg chain epitopes. This approach involves three different steps. First, the terminal Kdo sugar is activated with ADH by reductive amination of its ketone group, in the presence of NaCNBH_3_ as reducing agent, leading to the formation of O:2–ADH (**3,** **Figure 1A**). Second, O:2–ADH is further derivatized with adipic acid bis(N-hydroxysuccinimide) linker (SIDEA), introducing an active ester group on the Kdo (**4** **Figure 1A**, O:2–ADH–SIDEA). Finally, the reactive ester reacts with amino functionalities of CRM_197_ carrier protein, forming the selective conjugate O:2–ADH–SIDEA–CRM_197_ (**5,** **Figure 1A**). The resulting conjugate has a single attachment point of O:2 to the carrier protein.

In contrast, both random conjugation chemistries shown in **Figures 1B,C** introduce multiple attachment points along the OAg chain before linkage to the protein through oxidation with sodium periodate (NaIO_4_, **Figure 1B**) or CDAP chemistry (**Figure 1C**) (41). In the first case, oxidation of the vicinal diols of the polysaccharide with NaIO_4_ leads to the formation of aldehyde groups along the OAg chains, followed by their reductive amination with lysine residues of the protein in the presence of NaCNBH_3_ (O:2ox–CRM_197_ conjugate) (36). The oxidation step of O:2 involves both Rha and Glc residues that are the only sugar monomers having vicinal diols, resulting in the opening of the sugar rings. In the second strategy (**Figure 1C**), hydroxyl groups along the OAg chains are randomly activated through a cyanylating reaction performed by CDAP. CRM_197_ is directly added to the mixture without isolation of the intermediate activated polysaccharide, resulting in the formation of the O:2–CDAP–CRM_197_ conjugate (42). For both random strategies (e.g., oxidation/reductive amination and CDAP), CRM_197_ carrier protein is added directly to the activated polysaccharide without the need for protein derivatization or linker introduction on the OAg. **Table 1** reports characteristics of the three different conjugates obtained in terms of O:2-to-CRM_197_ ratio, free saccharide %, and free protein %. All chemistries preserved an O:2 OAc level close to 55%.

**Table 1:** Characterization of O:2–CRM_197_ produced with different conjugation chemistries.

Conjugate	O:2/CRM_197_ w/w ratio	Free saccharide %	Free protein %	OAc %
O:2–ADH–SIDEA–CRM_197_	0.79	2.2	4.5	56
O:2ox–CRM_197_	0.60	<5	Not detected	58
O:2–CDAP–CRM_197_	0.58	<5	<3.3	55

Between the two random conjugates, O:2–CDAP–CRM_197_ was selected for combination with Vi–CRM_197_ because it ensures improved protection of the OAg structural features. The CDAP chemistry avoids opening the sugar ring resulting from the oxidation step, which also makes the linkage of the oxidized sugar unit in the OAg chain weaker. The weaker oxidized sugar link is also associated with an overall reduction of the conjugated OAg size (36).

To compare the antibody responses induced by O:2–CDAP–CRM_197_ and O:2–ADH–SIDEA–CRM_197_ when formulated with Vi–CRM_197_, groups of 10 BALB/c mice were vaccinated SC on days 0 and 28 with corresponding bivalent formulations. Anti-O:2 and anti-Vi IgG responses in serum were measured after the first and second injection.

**Figure 2:**
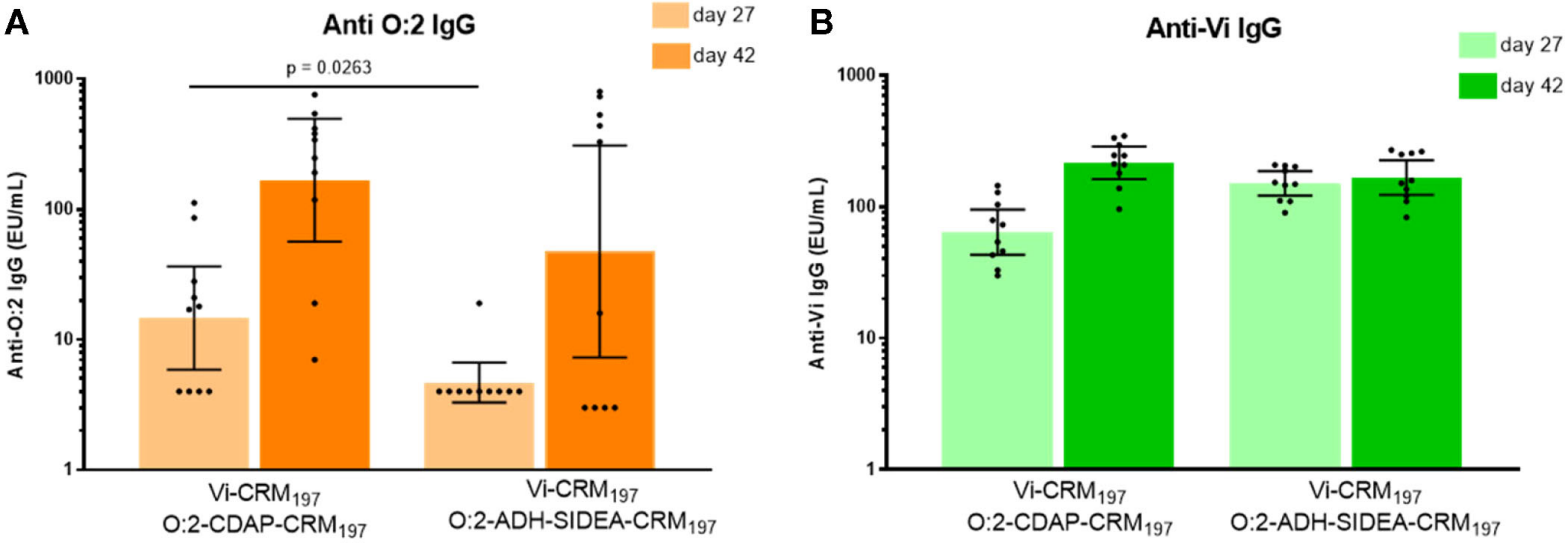
Immunogenicity study in mice testing bivalent formulations of Vi–CRM_197_ with O:2–CDAP–CRM_197_ or O:2–ADH–SIDEA–CRM_197_ in the presence of aluminum hydroxide. Balb/c mice (10 per group) were vaccinated SC on days 0 and 28 and sera were collected after one (day 27) and two injections (day 42). Specific IgG levels were determined by ELISA. Graphs of anti-O:2 **(A)** and anti-Vi **(B)** IgG geometric mean (bars) with 95% CI by group and individual animal antibody levels (dots).

The two bivalent formulations elicited similar anti-OAg IgG response on day 42 and anti-Vi IgG response on days 27 and 42 (**Figure 2**). The formulation with O:2–CDAP–CRM_197_ elicited significantly higher anti-O:2 IgG levels than the formulation with O:2–ADH–SIDEA–CRM_197_ on day 27 (*p* = 0.0263), while responses were similar on day 42.

**Table 2** summarizes the characterization of the two bivalent vaccine formulations over a period of 12 months at real-time storage conditions of 2–8 °C. For the formulation with O:2–ADH–SIDEA–CRM_197_, the free O:2 level increased from 6.6% to 22.7% within 3 months of storage and increased to 32.3% by 12 months (*p* = 0.026, linear regression slope). A much lower increase in free O:2 level was observed over 12 months of storage for the bivalent formulation prepared with O:2–CDAP–CRM_197_. Other stability-indicating data were similar between the two bivalent formulations over the 12-month period of analysis.

**Table 2:** Stability data collected on the bivalent formulations Vi–CRM_197_ + O:2–ADH–SIDEA–CRM_197_ and Vi–CRM_197_ + O:2–CDAP–CRM_197_ after storage at 2–8 °C up to 12 months.

Formulation	Test	Release	3 months	6 months	9 months	12 months	*p*-value**
O:2–ADH–SIDEA–CRM_197_+ Vi–CRM_197_	Vi content (μg)*	28	26	26	23	24	0.042
	O:2 content (μg)*	23	24	23	23	26	0.278
	CRM_197_ content (μg)*	67	62	60	60	71	0.752
	Free Vi %	8.5	8.9	14.4	16.4	15.7	0.031
	Free O:2%	6.6	22.7	21.7	30.1	32.3	0.026
	O-acetyl content (μmol)*	0.089	0.100	0.077	0.080	0.076	0.171
	pH	7.2	7.1	7.1	7.1	7.2	1.000
	Osmolality (mOsm/kg)	292	ND	ND	286	289	0.488
O:2–CDAP–CRM_197_+ Vi–CRM_197_	Vi content (μg)*	28	21	25	24	27	0.927
	O:2 content (μg)*	25	26	22	24	25	0.736
	CRM_197_ content (μg)*	80	81	78	81	82	0.485
	Free Vi %	8.6	14.7	13.6	11.7	12.4	0.607
	Free O:2%	<6.3	7.8	7.6	11.8	14.6	0.006
	O-acetyl content (μmol)*	0.093	0.064	0.080	0.084	0.073	0.639
	pH	7.5	7.4	7.5	7.5	7.51	0.491
	Osmolality (mOsm/kg)	328	329	326	nd	326	0.252

*Values refer to a sample volume of 0.5 mL. **Significance of the slope for linear regression: values below the quantification limits were replaced with half of the respective limit value.

The stability of O:2–ADH–SIDEA–CRM_197_, O:2–CDAP–CRM_197_, and Vi–CRM_197_ (as monovalent formulations) was further investigated under accelerated conditions (37 °C up to 4 weeks), focusing on the release of O:2 and Vi (**Table 3**). Results showed the stability of Vi–CRM_197_ and confirmed the higher stability of the random O:2 conjugate compared to O:2–ADH–SIDEA–CRM_197_. No aggregate formation was observed by HPLC-SEC in the formulation containing O:2–CDAP–CRM_197_ (**Supplementary Figure S1**), while aggregation was detected for the selective conjugate (**Supplementary Figure 1**). By performing similar studies on O:2ox–CRM_197_, the higher stability of this conjugate compared to O:2–ADH–SIDEA–CRM_197_ was also confirmed (**Table 3**; **Supplementary Figure 1**), suggesting that a chemistry involving the linkage of the carrier protein to the terminal Kdo could have been the cause of free saccharide release and aggregation.

**Table 3:** Release of free saccharide after storage of monovalent formulations at 37°C for up to 4 weeks.

Conjugate	Free saccharide increase %	
	Δ (*t*_14_ − *t*_0_)^*^	Δ (*t*_28_ − *t*_0_)^*^
O:2–ADH–SIDEA–CRM_197_	20	40
O:2–CDAP–CRM_197_	5	9
O:2ox–CRM_197_	4	7
Vi–CRM_197_	3	3

* Δ (*t_x_* − *t*_0_): difference in free saccharide % on day *x* vs. day 0.

Accelerated stability studies were conducted in the phosphate buffer pH 7.2.

### 3.2 Investigating the lability of the linkage of ADH to the Kdo terminal sugar of OAg

Initially, the reductive amination step for the synthesis of O:2–ADH (**3**, **Figure 1A**) was considered. Once verified that more than 90% of OAg chains were activated with ADH (by the TNBS colorimetric method) (35), the efficiency of the reduction performed with NaCNBH_3_ was investigated, hypothesizing the presence of the residual imino double bond O:2=ADH (**2,** **Figure 1A**) as a potential cause of release of free saccharide through the equilibrium, O:2=ADH → O:2 + ADH. The ability of NaCNBH_3_ to induce complete reduction of the double bond was evaluated by HPLC-SEC. In the absence of the reducing agent, alfa-ketohydrazone chromophore—characterized by a diagnostic UV absorption at 252 nm—is formed as a result of the addition of the hydrazide (ADH) to the carbonyl group of Kdo. Since no absorbance at 252 nm was observed at the level of the reductive amination intermediate (**Supplementary Figure S2**), the efficiency of the reduction was confirmed. As a result, the instability of the final conjugate O:2–ADH–SIDEA–CRM_197_ (5, **Figure 1A**) could not be attributed to the residual presence of the unreduced imino double bond of O:2=ADH (2, **Figure 1A**).

However, when the O:2–ADH (3) intermediate, isolated after the reductive amination, was incubated at 37 °C for 2 weeks at different pH values, the release of free linker was observed. Release of free linker was calculated as the difference between the free ADH measured on day 14 (*t*_14_) compared to time 0 (*t*_0_). A high release of free linker was observed especially at higher pH (**Table 4**). To better investigate the lability of ADH after its linkage to the Kdo terminal sugar of OAg (observed at the level of the first intermediate, 3, **Figure 1A**), different OAg–ADH constructs were synthesized starting from *Salmonella* Typhimurium (O:4,5) and *S.* Enteritidis OAg (O:9) (43), both sharing a terminal Kdo structure with O:2. Once stored at 37 °C for 14 days, both [O:4,5]–ADH and [O:9]–ADH also showed an increase of free linker at pH 9 (**Table 4**).

**Table 4:** Increase in free linker observed when purified OAg from different *Salmonella enterica* serovars (*S.* Paratyphi A, *S.* Typhimurium, and *S.* Enteritidis) and derivatized with ADH on the terminal Kdo sugar (by the selective reductive amination shown in **Figure 1A**) were stored at 37 °C.

Entry	Sample	Free linker increase Δ (*t*_14_ − *t*_0_) %				
		pH 5.0	pH 6.5	pH 7.2	pH 8.0	pH 9.0
1	*S*. Paratyphi A [O:2]–ADH	5.6	4.2	5.3	9.6	24.3
2	*S*. Typhimurium [O:4,5]–ADH	9.4	na	na	na	14.8
3	*S*. Enteritidis [O:9]–ADH	6.2	na	na	na	16.3

* Δ (*t_x_* − *t*_0_): difference in free saccharide % on day *x* vs. day 0.

Linker release was evaluated by HPLC-SEC after 14 days.

ESI MS (**Supplementary Figure S3B**) analysis conducted on the O:2–ADH sample after storage at 37 °C showed the presence of an adipic acid monohydrazide molecule. An internal nucleophilic attack of the carboxylic group of Kdo on the α-keto group of the hydrazide was hypothesized as a possible mechanism underlying the release of the linker (**Supplementary Figure S3A**). Based on the cumulative results, the free saccharide release observed in stability studies of O:2–ADH–SIDEA–CRM_197_ can be ascribed to the breakdown of the linkage between OAg Kdo sugar and ADH linker.

### 3.3 Immunogenicity in mice and rabbits of bivalent Vi–CRM_197_ + O:2–CDAP–CRM_197_ formulations obtained modulating aluminum hydroxide content

O:2–CDAP–CRM_197_ was selected for combination with Vi–CRM_197_, and different bivalent formulations were prepared for evaluation in mice or rabbits, by modulating the amount of aluminum hydroxide (**Table 5**) and verifying the impact its content can have on each conjugate adsorption and immunogenicity in animal models.

**Table 5:** Characterization of bivalent formulations differing for aluminum hydroxide content evaluated in mice and rabbits.

	Sample	Vi µg/mL	O:2 µg/mL	Aluminum hydroxide mg/mL Al^3+^	pH	Osmolality mOsmol/kg	% O:2 adsorption	% Vi adsorption
Mice	O:2 + Vi (unconjugated)	50	50	1.5	7.5	277	>90	>90
	O:2–CDAP–CRM_197_ + Vi–CRM_197_	25	25	0	7.5	323	na	na
				0.25	7.6	313	70.8	23.2
				0.75	7.6	297	66.9	69.7
				1.5	7.5	274	86.5	90.2
Rabbit	O:2–CDAP–CRM_197_ +Vi–CRM_197_	50	50	0	7.4	311	na	na
				0.25	7.5	303	58.0	12.9
				0.75	7.5	287	54.6	19.9
				1.5	7.5	262	62.7	42.3

When Vi and O:2 conjugates were formulated at 25 μg/mL each (for study in mice), by decreasing aluminum hydroxide content from 1.5 to 0.25 mg/mL, O:2–CDAP–CRM_197_ adsorption remained close to 70%, while Vi–CRM_197_ adsorption gradually diminished from 90% to 23%.

When co-formulating the two conjugates, each at a higher concentration of 50 μg/mL (for study in rabbits), and at the same concentration range of aluminum hydroxide, the maximum level of adsorption was lower than when half the amount of conjugates were used (62.7% vs. 86.5% for O:2 and 42.3% vs. 90.2% for Vi). Similarly, the adsorption of the O:2 conjugate was minimally impacted by the aluminum hydroxide content and remained between 50% and 60% in all formulations, while Vi conjugate adsorption again decreased with decreasing amounts of aluminum hydroxide, from 42% with 1.5 mg/mL aluminum hydroxide to 13% with 0.25 mg/mL aluminum hydroxide.

**Figure 3:**
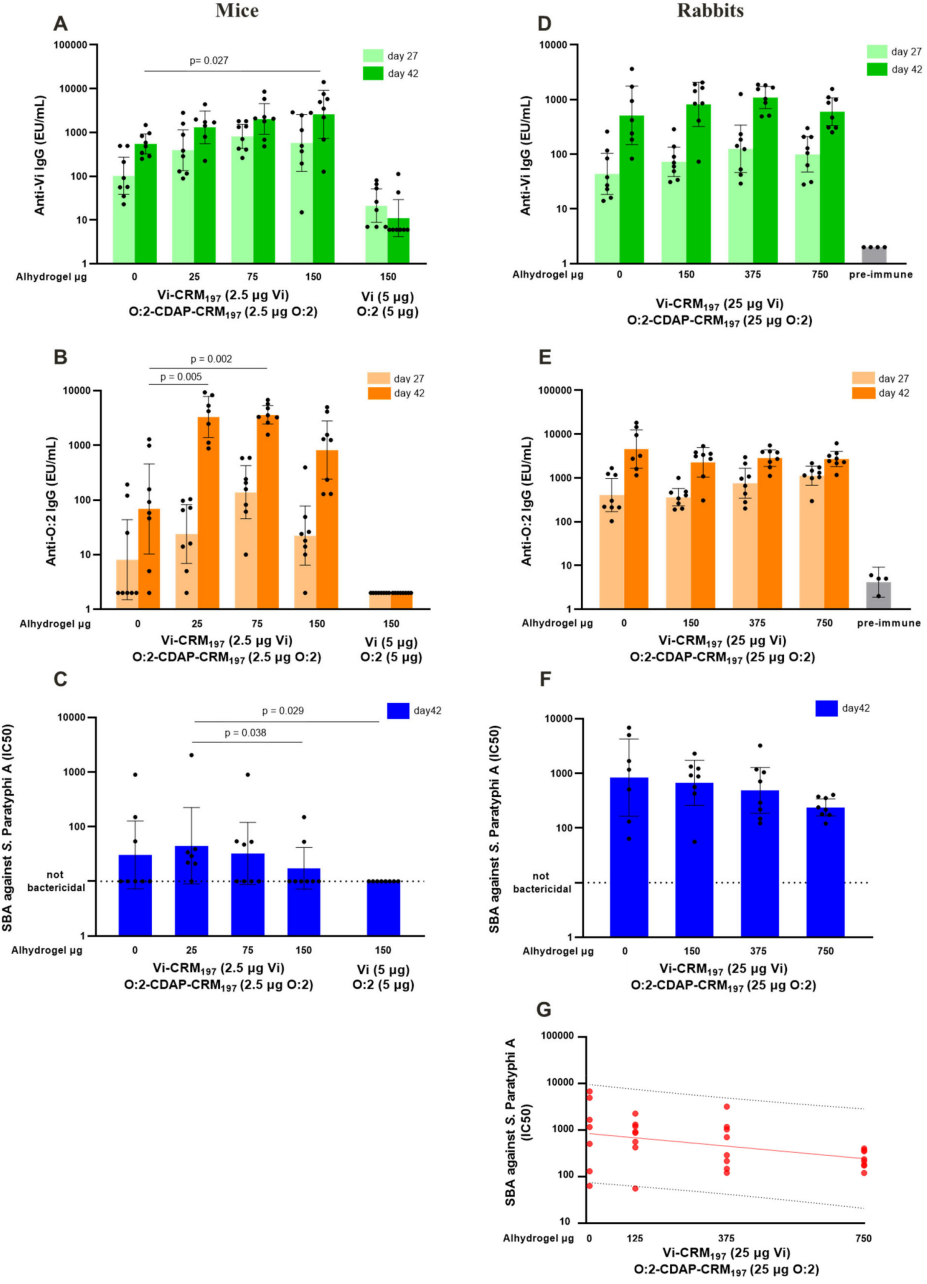
Results of immunogenicity studies in mice and rabbits evaluating bivalent formulations of Vi–CRM_197_ and O:2–CDAP–CRM_197_ differing in aluminum hydroxide content. All animals were vaccinated on day 0 and day 28: sera for antibody analysis were collected on days 27 and 42. Mice (eight per group) received 100 μL IP and rabbits (eight per group) received 500 μL IM of the indicated formulations. Graphs showing ELISA data by group geometric mean with 95% CI (bars) and individual animal EU/mL levels (dots) of anti-Vi total IgG on days 27 and 42 in mice **(A)** and rabbits **(D)**, and of anti-O:2 total IgG on days 27 and 42 in mice **(B)** and rabbits **(E)**. SBA data graphs showing the group geometric mean with 95% CI (bars) and individual animal IC_50_ levels against *S.* Paratyphi A wild-type strain on day 42 for the study in mice **(C)** and rabbits **(F)**. **(G)** Linear regression analysis for SBA IC_50_ vs. aluminum hydroxide dose with 95% CI from the study in rabbits. For comparison, data from eight mice vaccinated with unconjugated Vi and O:2 formulated on alum are shown in panels **(A–C)**, while data generated using pre-immune rabbit sera are shown in panels **(D, E)**.

In mice, all bivalent formulations induced anti-Vi and anti-O:2 IgG responses, higher than the corresponding unconjugated Vi and O:2 polysaccharides adsorbed on aluminum hydroxide, regardless of bleed day (**Figures 3A,B**). Only the conjugate formulation with an Al^3+^ content of 150 μg elicited statistically significant higher anti-Vi IgG on day 42 compared to the conjugate group without adjuvant (*p* = 0.027, Kruskal–Wallis with Dunn’s multiple comparison test, **Figure 3A**). The formulations containing 25 and 75 μg Al^3+^, but not 150 μg Al^3+^, elicited significantly higher anti-O:2 IgG response on day 42 than the unadjuvanted formulation (*p* = 0.005 and 0.002, respectively, Kruskal–Wallis with Dunn’s multiple comparison test, **Figure 3B**). In contrast to anti-O:2 ELISA values, bactericidal titers against *S.* Paratyphi A were low, with many non-responder animals (**Figure 3C**). Only the group receiving conjugates formulated with 25 μg Al^3+^ showed bactericidal titers significantly higher than SBA elicited by vaccination with unconjugated polysaccharide formulated on aluminum hydroxide (*p* = 0.029, Kruskal–Wallis with Dunn’s multiple comparison test) and the conjugates formulated with 150 μg Al^3+^ (*p* = 0.038, Kruskal–Wallis with Dunn’s multiple comparison test).

In rabbits, all bivalent conjugate formulations also elicited anti-Vi and anti-O:2 IgG responses (**Figures 3D,E**). For both conjugates, there was a strong antibody response to the polysaccharides after the first injection, which further increased after re-injection. The presence of aluminum hydroxide (in the 150–750 μg/dose range) did not impact either the anti-Vi or the anti-O:2 IgG response 27 days after the first and 14 days after the second immunization. Higher amounts of aluminum hydroxide in the formulation used for vaccination correlated with a decrease in the SBA titers against *S.* Paratyphi A (**Figure 3F**); linear regression analysis showed a decreasing trend of SBA with increasing doses of adjuvant (slope *p*-value 0.030). There was a 3.5-fold decrease in the SBA IC_50_ geomean between groups vaccinated with the formulation containing 750 μg/dose Al^3+^ and without aluminum hydroxide (**Figure 3G**).

## 4 Discussion

Multiple licensed vaccines are available for typhoid fever, while there are no licensed vaccines for *S.* Paratyphi A, and few bivalent vaccines against both *S.* Typhi and *S.* Paratyphi A are progressing through clinical trials. In this work, we combined the Vi–CRM_197_ conjugate (TYPHIBEV, Biological E) with a novel glycoconjugate against *S.* Paratyphi A. Different chemistries were tested for conjugation of O:2 to CRM_197_, the same carrier protein used in TYPHIBEV (26).

We initially explored a selective conjugation strategy targeting the terminal O:2 Kdo sugar, a theoretically attractive approach because it preserves the repeating-unit structure of the OAg and can avoid crosslinking, leading to a more defined and easier-to-characterize conjugate (35). However, the selective Kdo−based chemistry required multiple activation and purification steps, lowering process yields, and produced a final conjugate (O:2–ADH–SIDEA–CRM_197_) with inadequate stability and high levels of free polysaccharide. The quantity of free polysaccharide is a critical quality attribute for conjugate vaccines, as excessive free antigen can impair the T−helper cell-dependent anti−polysaccharide response (44, 45). Mechanistic characterization in our study linked the instability to a labile bond between Kdo and the ADH linker, explaining the release of free O:2.

Given these limitations, we evaluated alternative random chemistries (CDAP and periodate oxidation) that streamline manufacturing by reducing steps and intermediates. Importantly, O:2 conjugates prepared by random chemistries showed improved stability compared with O:2–ADH–SIDEA–CRM_197_, as hypothesized. We selected CDAP chemistry for development because it minimizes structural alteration of OAg compared with oxidation (which opens sugar rings) and because the CDAP method has been recently studied in depth to increase reproducibility and consistency, which can be weaker for random vs. selective (37). Notably, despite their different linkage patterns, the immunogenicity in mice of O:2–CDAP–CRM_197_ and O:2–ADH–SIDEA–CRM_197_ was comparable, indicating that the more manufacturable CDAP construct retained the relevant antigenic properties.

O:2–CDAP–CRM_197_ was therefore advanced into combination with Vi–CRM_197_ to form a bivalent vaccine candidate. We tested the bivalent formulation in mice and rabbits across a range of aluminum hydroxide concentrations to assess its adjuvant effect. The presence of aluminum hydroxide did not affect anti−Vi IgG responses in either species, indicating robust Vi immunogenicity independent of this adjuvant. These data confirm that there is no need for an adjuvant for the Vi component and indeed Vi conjugates are licensed without any adjuvant. In contrast, aluminum hydroxide modulated anti−O:2 responses in a species−dependent manner: it enhanced anti−O:2 IgG in mice, whereas in rabbits, higher aluminum hydroxide correlated with reduced bactericidal activity. In this study, mice developed low bactericidal titers, in contrast to previous published reports. A possible explanation is that a different immunization schedule was employed (two injections) compared with studies that observed bactericidal activity of mouse-derived antibodies after three injections (27, 35, 46). Fewer doses may have resulted in reduced antibody affinity maturation, limited class switching, or lower overall antibody titers, any of which could diminish functional activity. In rabbits, the inverse relationship between aluminum hydroxide concentration and SBA titers is difficult to interpret; while adsorption to alum can sometimes alter antigen presentation or stability (for example, by affecting O−acetylation), our measurements showed similar percentages of conjugate adsorption across formulations (**Table 4**), arguing against adsorption differences as the sole mechanism. These species−specific effects underscore the complexity of adjuvant–antigen interactions and the limitations of single−animal models to predict human immunogenicity. Because the impact of aluminum hydroxide on the human immunogenicity of this bivalent formulation could not be predicted with confidence from preclinical data, we prepared two clinical formulations (with and without aluminum hydroxide) and tested both in a Phase 1 study (NCT05613205). This exemplifies an adaptive, data−driven pathway to clinical testing that mitigates the risk of selecting a suboptimal formulation for first−in−human studies. Since licensed TCVs do not contain alum, avoiding an adjuvant would be preferable to simplify manufacturing. However, data collected to date do not show significant issues in formulation or characterization in the presence of alum, or any negative impact on the Vi-specific immune response.

Our work sits within a broader landscape of bivalent enteric fever vaccine development. Other groups are advancing Vi and O:2 combinations based on conjugation technology, using different carriers (e.g., Vi−TT plus O:2−DT; Vi−DT, and O:2−DT), or making use of alternative platforms (MAPS, live attenuated) (47). Beyond carrier choice, variables such as saccharide chain length, conjugation chemistry, saccharide loading, and adjuvant selection can all influence immunogenicity and functional antibody responses of resulting vaccine candidates. Clinical outcomes will be essential to understand how these manufacturing and formulation variables translate to protective efficacy.

In conclusion, in this work, we demonstrate that a selective Kdo−targeting approach, despite theoretical advantages for antigen definition, introduced process complexity, reduced yields, and generated a product with unacceptable stability. Switching to a CDAP random chemistry preserved antigenicity while markedly improving manufacturability and stability—attributes that are critical for scalable production and for meeting regulatory quality standards in vaccines destined for LMICs). This study supported the selection of O:2–CDAP–CRM_197_ for combination with Vi–CRM_197_. Our preclinical data informed a conservative and pragmatic clinical strategy (testing formulations with and without aluminum hydroxide), thereby minimizing development risk. If efficacious in humans, a bivalent Vi–O:2 conjugate vaccine would fill a major gap for the prevention of *S.* Paratyphi A disease and broaden protection against enteric fever caused by both *S.* Typhi and *S.* Paratyphi A. Wider deployment could substantially reduce morbidity and mortality and help to curb antibiotic use and the emergence of resistance in endemic settings.

## Data Availability

The original contributions presented in the study are included in the article/**Supplementary Material**. Further inquiries can be directed to the corresponding author.
