# Supplementary material for: Preclinical evaluation of a bivalent conjugate vaccine against Salmonella Typhi and Paratyphi A
Source: Front Immunol. 2026 May 1;17:1726455. doi: 10.3389/fimmu.2026.1726455 (PMC13176246; doi:10.3389/fimmu.2026.1726455)
Supplement: Supplementary file 1 [file DataSheet1.pdf]

# *Supplementary Material*

**A) O:2-ADH-SIDEA-CRM$_{197}$**

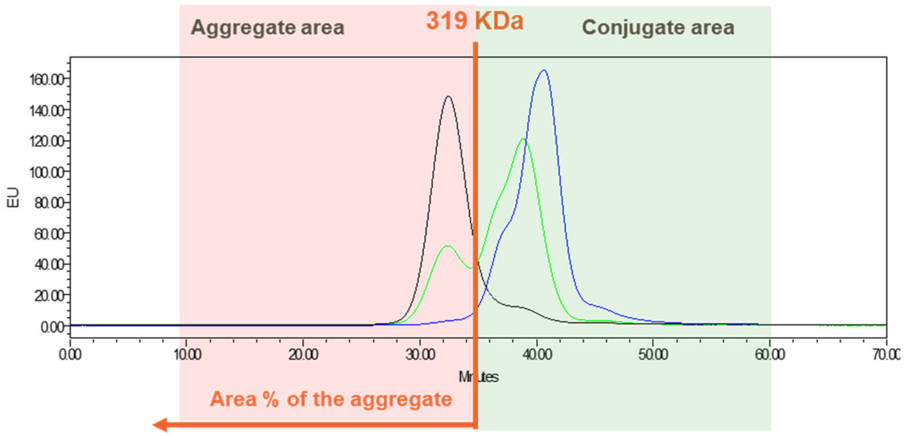


**B) O:2-CDAP-CRM$_{197}$**

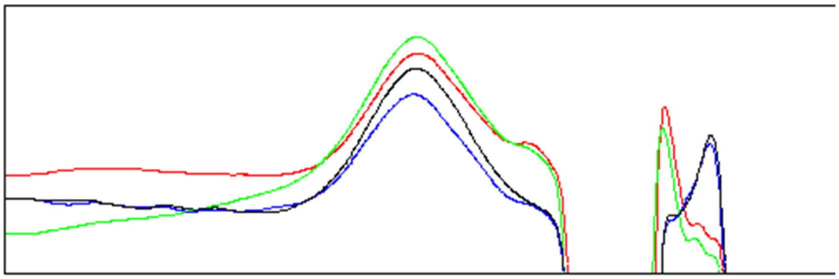

**C) O:2ox-CRM197**

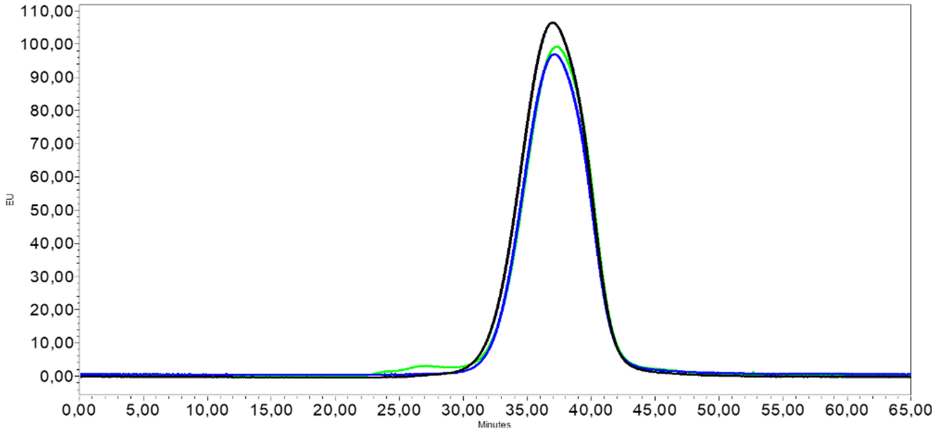

**Figure S1.** A) HPLC-SEC profiles (fluorescence emission detection ex280/em336 nm; TosoH TSK gel 6000+5000 PW columns connected in series) of O:2-ADH-SIDEA-$CRM_{197}$ conjugate showing the increase of the area above MW 319 kDa when aggregation was present. Original conjugate with no aggregation (blue line), partial aggregation (green line) and complete aggregation (black line). B) HPLC-SEC profiles (Refractive index; TosoH TSK gel 6000+5000 PW columns connected in series) of O:2-CDAP-$CRM_{197}$ conjugate (CDAP chemistry) registered at $t_0$ (black) and after storage at 37 °C for 8 days (blue), 14 days (green) and 28 days (red). The overlapping of all chromatographic profiles at the different timepoints demonstrated that aggregation didn't occur for this type of chemistry during the accelerated stability. C) HPLC-SEC profiles (fluorescence emission detection ex280/em336 nm; TosoH TSK gel 6000+5000 PW columns connected in series) of O:2ox-$CRM_{197}$ conjugate (reductive amination chemistry) registered at time zero ($t_0$, black line) and after storage at 37 °C for 14 days (blue) and 28 days (green). The chromatographic profiles overlapping demonstrated that aggregation didn't occur during the accelerated stability.

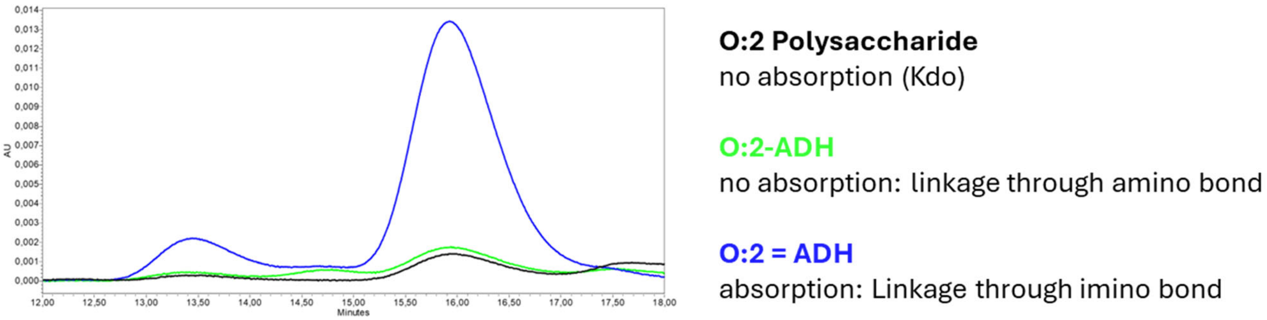


**Figure S2.** HPLC-SEC profiles (absorption at 252 nm; TosoH 3000PW-XL) of O:2, O:2-ADH and O:2=ADH.

A)

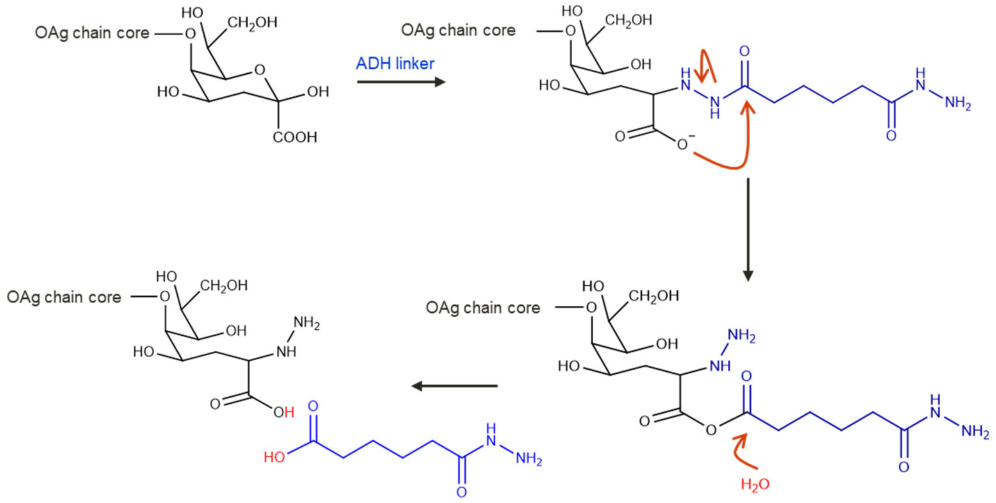


B)

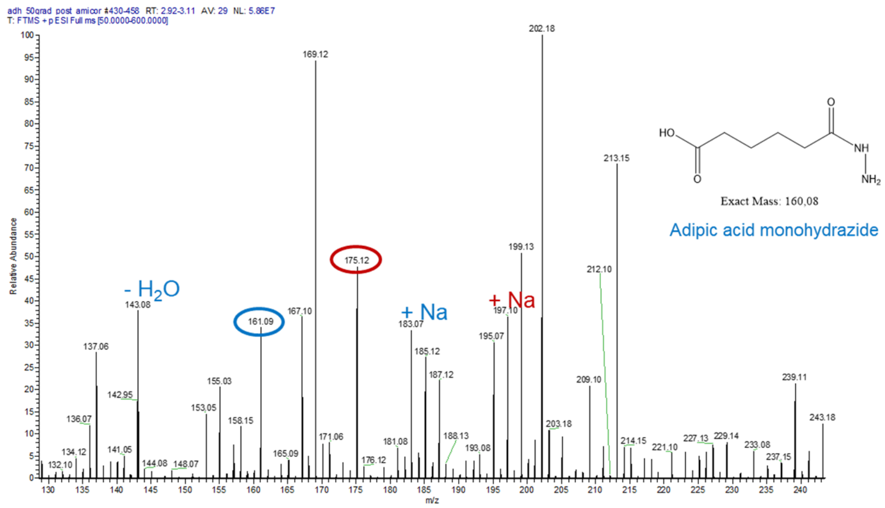


**Figure S3.** A) Possible mechanism of the breakage between O:2 and $CRM_{197}$ at the level of Kdo linkage. Instability involving an internal attack of the carboxylic group on the hydrazide NH-CO of Kdo. B) ESI Mass analysis (infusion) of small molecules released from a sample of O:2-ADH after accelerated stability, with the detection of the adipic acid monohydrazide expected based on the mechanism hypothesized.
